# Apoptosis Therapy in Cancer: The First Single-molecule Co-activating p53 and the Translocator Protein in Glioblastoma

**DOI:** 10.1038/srep04749

**Published:** 2014-04-23

**Authors:** Simona Daniele, Sabrina Taliani, Eleonora Da Pozzo, Chiara Giacomelli, Barbara Costa, Maria Letizia Trincavelli, Leonardo Rossi, Valeria La Pietra, Elisabetta Barresi, Alfonso Carotenuto, Antonio Limatola, Anna Lamberti, Luciana Marinelli, Ettore Novellino, Federico Da Settimo, Claudia Martini

**Affiliations:** 1 Department of Pharmacy, University of Pisa, Pisa, Italy; 2 Department of Clinical and Experimental Medicine, University of Pisa, Italy; 3 Department of Pharmacy, University of Naples Federico II, Italy; 4 Dipartimento di Scienze Motorie, University of Naples “Parthenope”, Naples, Italy

## Abstract

In the complex scenario of cancer, treatment with compounds targeting multiple cell pathways has been emerging. In Glioblastoma Multiforme (GBM), p53 and Translocator Protein (TSPO), both acting as apoptosis inducers, represent two attractive intracellular targets. On this basis, novel indolylglyoxylyldipeptides, rationally designed to activate TSPO and p53, were synthesized and biologically characterized. The new compounds were able to bind TSPO and to reactivate p53 functionality, through the dissociation from its physiological inhibitor, murine double minute 2 (MDM2). In GBM cells, the new molecules caused Δψm dissipation and inhibition of cell viability. These effects resulted significantly higher with respect to those elicited by the single target reference standards applied alone, and coherent with the synergism resulting from the simultaneous activation of TSPO and p53. Taken together, these results suggest that TSPO/MDM2 dual-target ligands could represent a new attractive multi-modal opportunity for anti-cancer strategy in GBM.

Combination therapy has been the standard of care in several diseases, such as diabetes and immunoinflammatory disorders^1^, since it is a rational strategy to decrease drug resistance and to increase response and tolerability.

The advantages of multi-target action are well known in cancer, as oncogenesis is a multigenic, multifactorial process, characterized by the misregulation of more than one protein^2^. Conventional chemotherapeutic agents are currently administered as co-therapies, while a new class of receptor tyrosine kinase (RTK) inhibitors with multi-target action in a single chemical entity have entered the market or are in clinical development^3^.

The multi-target strategy may be of great importance against glioblastoma multiforme (GBM), a particularly aggressive form of brain malignancy. Despite massive research efforts, the surgical resection and the currently approved anti-GBM agents, such as Temozolamide, and Bevacizumab, or Cilengitide, which is in clinical trials (http://clinicaltrials.gov), offer a limited improvement in progression free survival^4^,^5^,^6^. Escape from cell death is a hallmark of cancers and a major cause of treatment failure; in this respect, apoptosis inducers that act through the mitochondria death pathway have been emerging as promising drugs in a large number of tumors^7^, particularly in GBM^8^. Activation of the cancer cell death machinery through the mitochondrial membrane permeabilization has been obtained so far also by the use of drugs targeting the mitochondrial translocator protein (TSPO). TSPO activators such as PK11195, Ro5-4864 and diazepam, have demonstrated anticancer effects *in vitro* and *in vivo*, both as single agents or combined with the chemotherapeutic agents etoposide or ifosfamide^9^,^10^. Importantly, in several cellular or animal models, PK11195 has demonstrated to reduce or abrogate the antiapoptotic effect of Bcl-2-family proteins, suggesting that TSPO may be used to bypass Bcl-2-imposed chemoresistance^9^. In this respect, we have recently demonstrated that newly synthesized selective TSPO ligands are able to trigger apoptosis also in human GBM cell lines and in rat C6 glioma cells, modulating the opening of the mitochondrial permeability transition pore (MPTP), of which TSPO is an important constitutive protein^11^,^12^.

Another important role in mitochondria-mediated cell apoptosis involves the tumor suppression protein p53. Indeed, in addition to target gene regulation, p53 can directly induce permeabilization of the outer mitochondrial membrane, by forming complexes with the protective Bcl2-family proteins, resulting in cytochrome c release^13^. The deregulation of this pro-apoptotic protein is widely described in literature, and the reactivation of its endogenous function represents an important anti-cancer therapeutic strategy^14^, at least for those tumors with a no mutant p53. P53 is negatively regulated by the murine double minute 2 (MDM2), and thus inhibitors of MDM2/p53 interaction currently represent another viable approach in GBM therapy^14^,^15^,^16^.

Thus, while agents targeting either the TSPO or MDM2/p53 interaction have been already investigated and provided to have some survival benefit in cancers, molecules targeting both proteins are not known so far. In this work, with the perspective that co-targeting TSPO and MDM2/p53 with one molecule could maximize the anti-tumor efficacy, dual binders were rationally designed, synthesized and biologically characterized in human GBM cells. Specifically, molecular modeling studies were firstly conducted to identify, among our in-house database of TSPO ligands^11^,^12^,^17^,^18^,^19^, those that, by adequate decoration, would disrupt the p53/MDM2 interaction, while retaining the TSPO binding activity. Our approach was successful leading to the 2-phenylindolylglyoxylyldipeptides **1** and its acid analogue **7**. These molecules, binding TSPO and disrupting MDM2/p53 interaction, caused MPTP opening, and transmembrane mitochondrial potential (ΔΨm) dissipation. These events resulted in cell-cycle arrest, apoptosis and a final anti-proliferative effect on human GBM cells.

These effects were similar to those obtained by coincubation with a typical TSPO ligand and a MDM2/p53 inhibitor, namely PK11195 and Nutlin-3, respectively.

To the best of our knowledge, this is the first work on TSPO-MDM2 dual-target ligands. These agents represent an attractive multi-modal opportunity for enhancing anti-cancer activity in a tumor with poor prognosis, such as GBM.

## Results

### Design and synthesis

**Figure 1:**
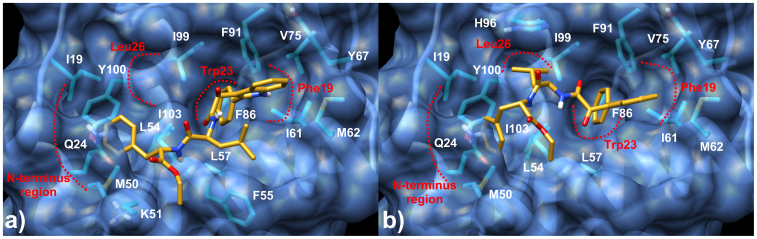
Docking poses of Compound 1 in the MDM2 binding site. The ligand is represented as golden stick, the protein residues and surface as cyan sticks and transparent blue respectively. MDM2 binding pockets are labeled according to p53 side chains and are highlight in red dots.

**Figure 2:**
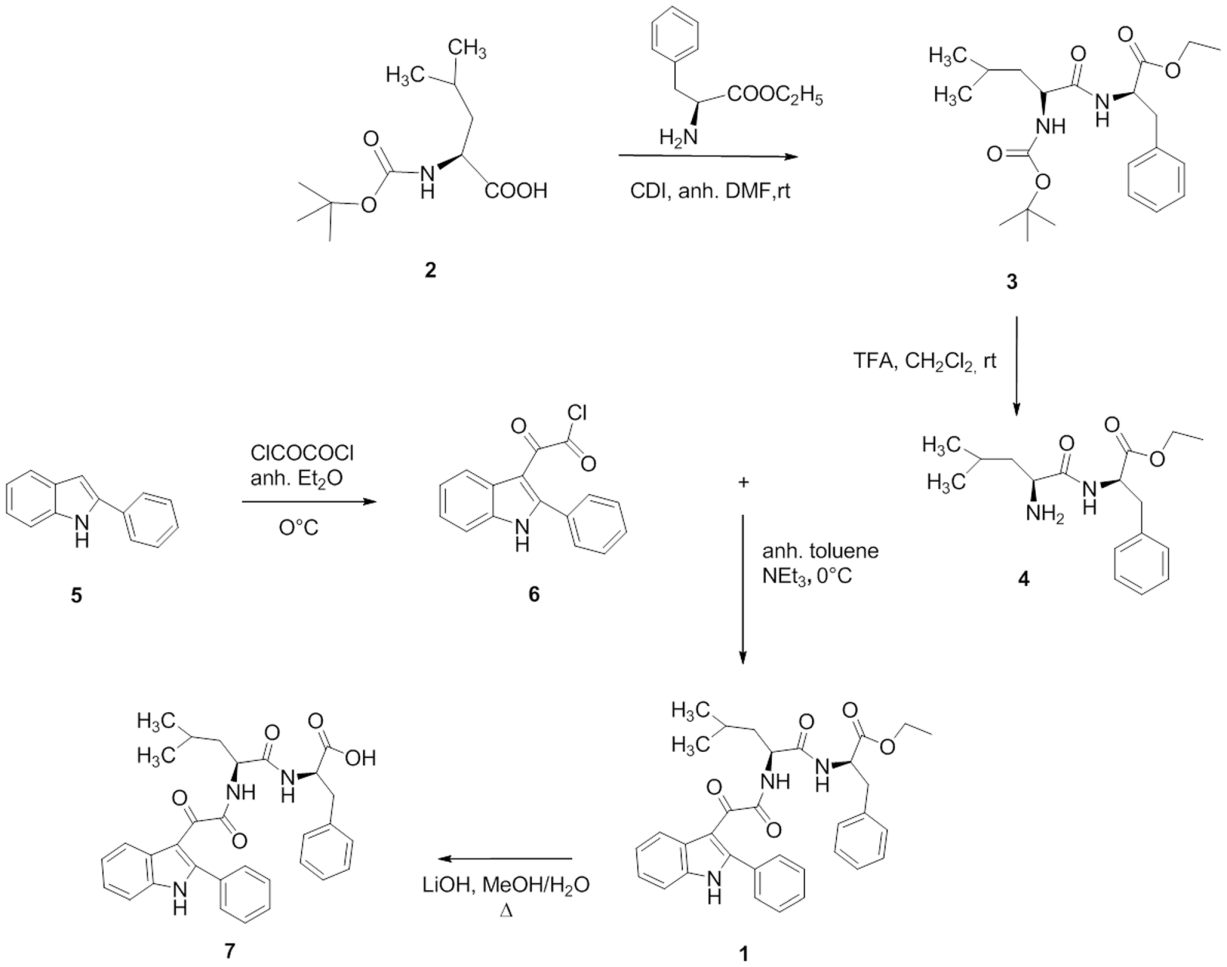
Scheme of synthesis of novel indolylglyoxylyldipeptides.

With the aim to target TSPO and MDM2/p53 at the same time, molecular modeling studies were conducted to identify, among our in-house database of TSPO ligands^11^,^12^,^17^,^18^,^19^, those that would disrupt the p53/MDM2 interaction if adequately decorated. Among a number of chemically diverse structures, we focused on indole-containing structures, as indole is considered a “privileged scaffold” for drug discovery^20^. Specifically, we focused on phenylindolylglyoxylamides (PIGA), which are prone to be easily substituted (eg. on the amide nitrogen) and for which an exhaustive SARs study has already been performed by our group providing a clear picture of PIGA-TSPO interaction^17^,^18^,^21^. PIGA SARs clearly suggested the essential requirements for binding to TSPO: (i) the presence of one H-bond acceptor (the oxygen atom of the second carbonyl group of the oxalyl bridge); (ii) an aromatic moiety at the 2-position of the indole nucleus; (iii) N,N-disubstitution at the amide nitrogen. Herein, bearing in mind all this information, we decided to substitute the alkyl groups with chemical features mimicking the p53 trans-activation domain helix. Thus, for the design of the novel MDM2 inhibitors, both crystallographic data, revealing the mode of binding of p53/MDM2, and the MDM2 ligands reported so far^22^ were taken into account. As shown in Suppl. Figure 1, the three critical residues of p53 transactivation domain are bound to MDM2 N-terminal domain in a well-defined surface pocket characterized by three hydrophobic clefts hereon named: Phe19, Trp23 and Leu26 pockets. Very recently, new MDM2 “hot spots” for the development of potent inhibitors have been available and suggested that the MDM2 N-terminus region, which is a disordered loop in the apo-structure, can adopt an ordered folding and establish additional van der Waals and hydrogen bond interactions upon the binding of an inhibitor, considerably augmenting the activity of the last^23^. Considering all this information, we attempted, as first step, the docking calculation of unsubstituted 2-phenylindolyglyoxylamide in the MDM2 pocket, through the Autodock 4 program. In the lowest energy binding pose, the phenyl ring was buried in the Trp23 pocket, the indole ring occupied the Phe19 region, while the glyoxylamide moiety was oriented toward the Leu26 pocket offering chances for a further decoration. Thus, similarly to p53 transactivation domain helix, we functionalized the glyoxylylamide group with a Leu residue. Subsequently, we decided to further elongate our compound in the attempt to reach the region nearby the MDM2 N-terminus. In fact, residue Leu26 of p53 is followed by a proline residue which in the 1YCR X-ray structure interacts with the MDM2 Y100. In order to mimic and enhance such hydrophobic contact, we decided to add a Phe residue. The design strategy resulted in compounds **1** and **7** (Figures 1 and 2).

As proof of concept of the design hypothesis, docking calculation of **1** and **7** in the MDM2 binding site was attempted and two main binding poses were found in the lowest energy clusters.

In particular, in the first one (hereon referred as binding mode A, Figure 1a) the ligand was inserted in the MDM2 binding site so that the phenyl substituent bound to the indole ring was buried in the Trp23 pocket making favorable van der Waals contacts with L57, I61, F86, F91, I99 and I103 side-chains. The MDM2 Phe19 subpocket was occupied by the indole core that was able to make hydrophobic contacts with I61, M62, V75 and Y67 residues. On the other side of **1**, Phe residue was accommodated within the MDM2 N-terminus region where it formed hydrophobic contacts with I19, Y100, L54 and M50 and engaged an amide-π interaction with the Q24 side chain, while the ethyl ester moiety was found sandwiched between F55 and the methylene groups of the K51 side chain. Oddly, Leu26 cleft was not filled by the Leu side chain of **1** that, instead, establishes hydrophobic interactions with F55 residue. Accordingly, in a very recent work by Olson's group the benzyl moiety of their morpholinones ligands engaged an edge-face π−π interaction with the same F55 leaving Leu26 subpocket unoccupied just as in our case^24^. However, the occupancy of Leu26 pocket by the Leu residues of **1** cannot be strictly excluded. In fact, in the energetically equivalent binding mode B (Figure 1b), the inhibitor was positioned so that the isobutyl side chain was well inserted into Leu26 subpocket establishing lipophilic contacts with H96, I99 and Y100 residues. Notably, in the case of **7**, (where the binding mode B is preferred) docking results showed that the carboxylic group flipped toward the Leu26 pocket H-bonding the Nδ of the H96 imidazole ring, thus compensating the loss of the hydrophobic interactions established by the ethyl ester **1**. In both cases, the found binding modes definitely suggested that the design hypothesis was sound and that the synthesis of **1** and **7** was worthwhile.

The convergent procedure applied fort the synthesis of the target indole derivative **1** is outlined in Figure 2. The dipeptide **4** was obtained in two steps starting from the Boc-L-leucine **2** that was condensed with L-phenylalanine ethyl ester, in the presence of N,N′-carbonyldiimidazole (CDI) in anhydrous dimethylformamide to obtain compound **3**, and then deprotected by treatment with trifluoroacetic acid in dichloromethane. Acylation of the commercially available 2-phenylindole **5** with oxalyl chloride, in anhydrous ethyl ether at 0°C, yielded the corresponding 2-phenylindolylglyoxylyl chloride **6**^17^,^18^. Reaction of derivative **6** with compound **4**, in the presence of triethylamine in anhydrous toluene at 0°C, gave compound **1** which was purified by flash chromatography. The ester moiety of **1** was finally hydrolyzed with lithium hydroxide monohydrate in a MeOH/H_2_O (3:1) solution under reflux overnight to yield the corresponding carboxylic acid **7**.

### TSPO activity in “cell-free” models

**Figure 3:**
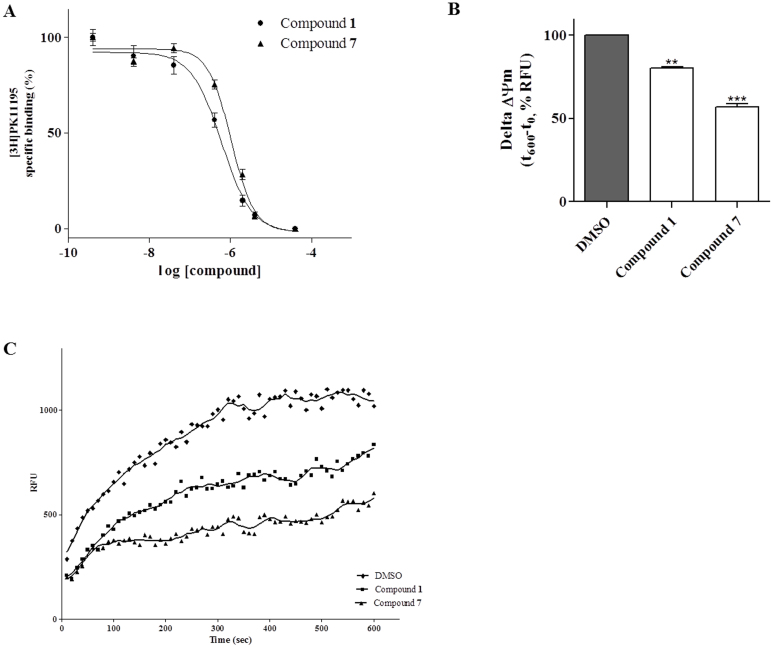
New synthesized compounds bind to TSPO and induce Δψm collapse. (A) *[*^*3*^*H]PK11195 radioligand binding:* membranes homogenates obtained from kidney (20 μg of proteins) were incubated with 0.6 nM [^3^H]PK11195 and different compounds concentration. Reaching equilibrium, samples were filtered and bound radioactivity was counted. Data are expressed as percentage of specific binding versus basal value (set to 100%) and represent the mean ± SEM of three different experiments. (B) *Evaluation of Δψm in isolated mitochondria:* Mitochondria (5 μg of proteins) were treated with compound **1** (5 μM), or compound **7** (5 μM), or DMSO (control) for 15 min. The Δψm was evaluated using JC-1 protocol as describe in *Methods*. The data are expressed as variation of JC1 uptake into mitochondria, calculated as the difference between RFU read after 10 minutes and RFU read at the beginning. Data represent the mean ± SEM of three different experiments. Each experiment was performed in duplicate. Statistical significance was determined with a one-way ANOVA with Bonferroni post-test:**P < 0.01, ***P < 0.001 vs control. (C) Representative graph of mitochondria potential evaluation using JC-1 protocol. The results were expressed as RFU units in time.

At first, the ability of the novel compounds **1** and **7** to bind TSPO was tested. For this purpose, radioligand binding assays with the TSPO-selective radioligand [^3^H]PK11195 were performed. Compounds **1** and **7** were able to displace specific [^3^H]PK11195 binding, in a concentration-dependent manner, with Ki values of 438 ± 35 nM and 759 ± 56 nM (Figure 3A), respectively.

Several evidences have shown that TSPO is a constituent of the MPTP, taking therefore part in dissipation of Δψm that occurs after MPTP opening, as well as in the release of proapoptotic inter-membrane proteins and in the induction of apoptosis^25^. To assess whether compounds **1** and **7** could modulate MPT-pore opening through their selective binding to TSPO, Δψm was measured in mitochondria isolated from the human GBM cell line U87MG. The treatment of the isolated mitochondria with compound **1** or **7** (5 μM) for 15 min lead to a significant reduction in tetrachloro-tetraethylbenzimidazolylcarbocyanine iodide (JC-1) accumulation compared to control untreated mitochondria, demonstrating the collapse of Δψm (Figures 3B and C).

### Human MDM2/p53 complex dissociation in “cell-free” models

The ability of the novel compounds to dissociate the MDM2/p53 complex was investigated by NMR and ELISA-based *in vitro* assays on recombinant and native human MDM2/p53 complex, respectively.

**Figure 4:**
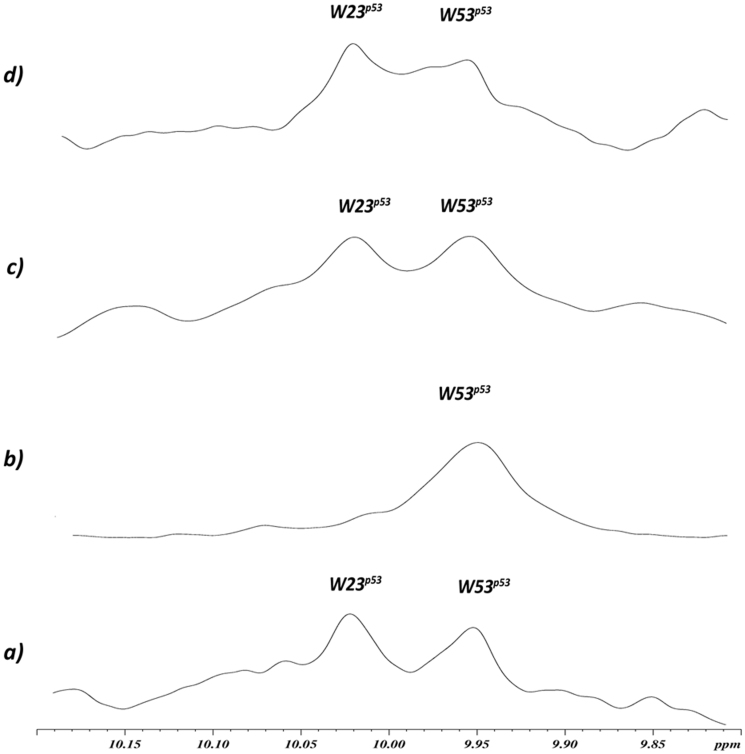
Dissociation of recombinant MDM2/p53 complex by NMR studies. One-dimensional proton spectrum of the side chains of tryptophans (W) of p53 alone (a), MDM2/p53 complex (b), MDM2/p53 complex after addition of compound **7** (c), or compound **1** (d). Figure shows the ^1^H NMR signals of the tryptophan residues of p53 and of MDM2/p53 complex (0.1 mM, only W53 ^N^H^ε^ side chains signal can be detected in the last). After the addition of compound **7** or **1** (0.2 mM formal final concentration) to the MDM2/p53 complex, the W23 peak appears (c and d, respectively) indicating a complete p53 release.

Holak et al. have recently developed an NMR assay to determine the ability of antagonists to dissociate protein-protein complexes. The method, named AIDA (for Antagonist Induced Dissociation Assay^26^,^27^), can work with complex made by a large protein fragment (larger than 30 kDa) and a small reporter protein (less than 20 kDa). AIDA makes use of two-dimensional^15^N-HSQC spectra, however, in the presence of flexible protein residues, 1D proton NMR spectra may be sufficient for monitoring the states of the complex upon addition of ligands. Since the N-terminal domain of p53 is highly flexible, MDM2/p53 complex is suitable for 1D proton NMR application^26^,^28^,^29^. Particularly, the signals from ^N^H^ε^ side chains of W23 and W53 are sharp in the free p53 1D proton spectrum. After the complex formation, W23 signal disappears, since W23 comprises the primary binding site for MDM2. In fact, residues 17–26 of p53 participate in well-defined structures of large MDM2/p53 complexes. In contrast, W53 is still not structured when p53 binds MDM2^26^. Due to the reduced flexibility in the complex, the observed 1/T2 transverse relaxation rate of the bound W23 increases considerably, leading to the broadening of NMR resonances and in the disappearance of this signal from the spectra.^1^H NMR signals of the tryptophan residues of p53 and of MDM2/p53 complex are shown in Figure 4 (0.1 mM, only W53 ^N^H^ε^ side chains signal can be identified in the last). When compound **1** or **7** are added (0.2 mM formal final concentration) to the MDM2/p53 complex, the W23 peak appears (Figure 4C and Figure 4D, respectively) signifying a complete p53 release^26^.

**Figure 5:**
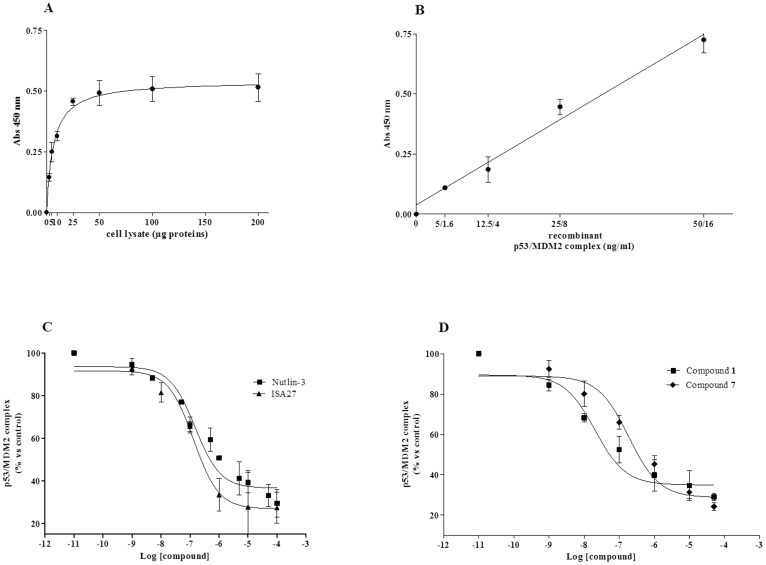
ELISA-based *in vitro* MDM2/p53 protein-protein interaction assay. (A), (B) Different amount of cell lysates (panel A) or human recombinant MDM2/p53 protein (panel B) were captured on wells pre-coated with MDM2 antibody. After extensive washes, levels of the MDM2/p53 complex were quantified using an antibody specific for p53, and subsequently an HRP-conjugated antibody and a TMB substrate kit. (C), (D) U87MG cell lysates, containing the native MDM2/p53 complex, were pre-incubated with DMSO (control) or different concentrations of the indicated compounds. Then, lysates were captured on wells pre-coated with MDM2 antibody. After extensive washes, levels of the MDM2/p53 complex were quantified using an antibody specific for p53, and subsequently an HRP-conjugated antibody and a TMB substrate kit. Blank wells were obtained in the absence of p53 antibody. Data are expressed as absorbance at 450 nm minus blank values (A), (B), or as % of control set to 100% (C), (D), and represent the mean ± SEM of at least three independent experiments.

To confirm the qualitative results obtained in the NMR experiments, a quantitative sandwich immune-enzymatic assay technique, on crude cell lysates obtained from U87MG cells was developed. As a first step, protein dependence of the assay was revealed by adding increased aliquots of U87MG cell lysate. As shown in Fig. 5A, specific absorbance values at 450 nm proportionally increased with protein concentration of U87MG cell lysates, with a trend toward hyperbole saturation starting from 40 μg of proteins. The absorbance at 450 nm of blank wells, obtained in the absence of p53 antibody, remained always under the 20% of total values. The assay was also validated using different concentration of human recombinant MDM2/p53 complex (Figure 5B). As reference compounds, the two characterized MDM2 inhibitors, Nutlin-3 and ISA27 were used^16^,^29^. As depicted in Figure 5C, both Nutlin-3 and ISA27 dissociated the MDM2/p53 complex, with IC_50_ values of 108.0 ± 4.5 nM and 121.7 ± 14.5 nM, respectively. These values are comparable to those obtained using Biacore's surface plasmon resonance technology^30^, or a similar ELISA assay on recombinant MDM2/p53 proteins^31^. Compounds **1** and **7** were thus tested with the validated ELISA assay, showing to be able to efficaciously dissociate MDM2/p53 complex, with IC_50_ values in the nanomolar range (Figure 5D). Noticeably, compound **1** appeared to be approximately 10 times more potent than the reference MDM2 inhibitors Nutlin-3 or ISA27, with an IC_50_ value of 11.65 ± 0.49 nM, whereas compound **7** showed similar potency to the reference MDM2 inhibitors (IC_50_ value of 202.0 ± 21.2 nM).

### MDM2/p53 complex dissociation and reactivation of p53 function in U87MG cells

The cellular parameters commonly studied to support the reactivation of p53 pathway, following cell exposure with a MDM2 inhibitor^15^,^16^,^30^, were then evaluated. First, we examined the effect of compounds **1** and **7** on p53 protein levels in U87MG cells, a cell line widely used as a representative model of GBM. These cells maintain wild-type p53, and show reduced p53 functions due to the overexpression of MDM2^32^.

**Figure 6:**
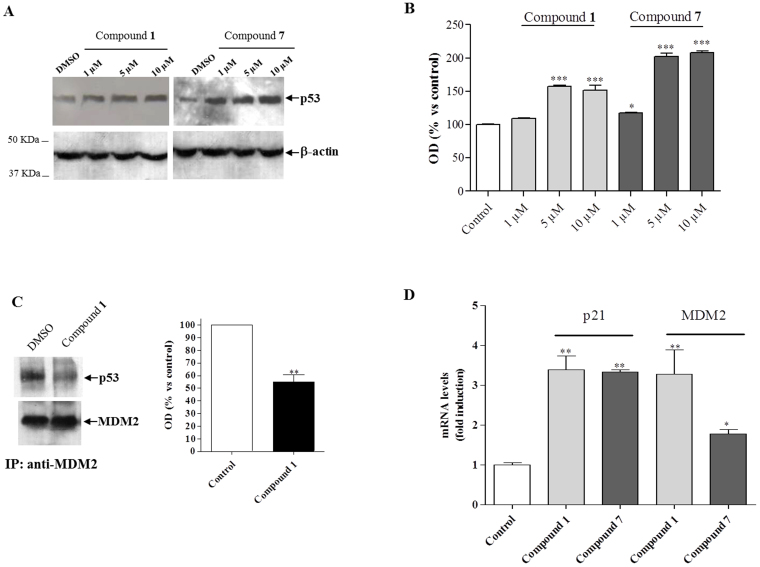
New synthesized compounds induce p53 protein accumulation by dissociation of MDM2/p53 complex and stimulate transcription of p53 target genes in U87MG cells. The U87MG cells were treated with DMSO (control sample) or the indicated concentrations of the compound **1** or **7** for 12 h. Lysates were subjected to Western blot analysis using antibody to p53 (FL-393; Santa Cruz Biotechnology). One representative Western blot is presented (panel (A) for each cell treatment. β-actin was used as the loading control. The bar graph (panel (B) shows the quantitative analysis of the Western blots, performed using ImageJ. Data are expressed as the percentage of OD versus control set to 100% and represent the mean ± SEM of three different experiments. Statistical significance was determined with a one-way ANOVA with Bonferroni post-test: *P < 0.05, ***P < 0.001 vs Control; (C) *Evaluation of MDM2/p53 complex:* U87MG cells were incubated with compound **1** (5 μM) for 8 h followed by immunoprecipitation using an anti-MDM2 antibody. The MDM2/p53 complex and the relative input of the proteins were detected by immunoblot. One representative Western blot is presented (left panel). The bar graph (right panel) shows the quantitative analysis of the Western blot, performed using ImageJ. Data represent the mean ± SEM of three different experiments. **P < 0.01 vs Control. Full-length blots are reported in Supplementary Information section titled “Full-length blots relative to the cropped images showed in the main Figures”. (D) *Relative mRNA quantification of p53 target genes:* The relative mRNA quantification of p53 target genes (p21 and MDM2) was performed by real-time RT-PCR as describe in *Methods*. Data represent the mean ± SEM of three different experiments. Each experiment was performed in duplicate. Statistical significance was determined with a one-way ANOVA with Bonferroni post-test: *P < 0.05, **P < 0.01 vs Control.

The incubation of the U87MG cells with compounds **1** or **7** for 8 h caused an increase in p53 protein levels (Figure 6A and B). The accumulation of p53 was dose-dependent, and became significant at 5 μM concentration of the tested compounds (Figure 6A and B).

To confirm the ability of compound **1** to disrupt the intracellular MDM2/p53 interaction, a co-immunoprecipitation assay was used. Specifically, MDM2 was immunoprecipitated from U87MG cells treated with DMSO or 5 μM compound **1**, and then a western blot analysis on MDM2 immunoprecipitates was performed using a specific antibody against p53. As depicted in Figure 6C, only minimal amounts of p53 could be detected in MDM2 immunoprecipitates after 8 h of cell treatment with 5 μM compound **1** as compared with control cells. These results indicated that the compound **1**-induced p53 accumulation was due to a reduced interaction between p53 and MDM2.

Furthermore, compounds **1** and **7** were demonstrated to increase significantly the mRNA levels of p53 target genes in U87MG cells (i.e. MDM2 and the cyclin-dependent kinase inhibitor p21) (Figure 6D).

### Cell cycle block and apoptosis activation in U87MG cells

**Figure 7:**
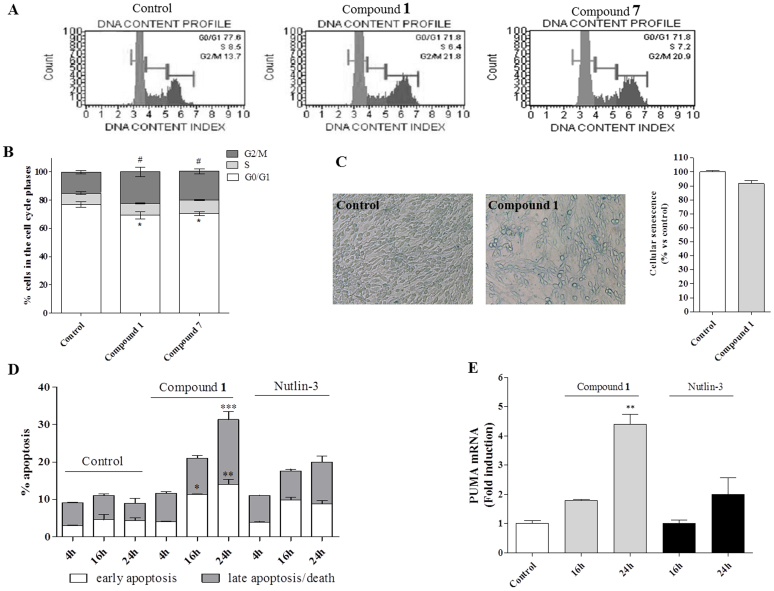
New synthesize compounds trigger checkpoint-dependent cell-cycle arrest and apoptosis in U87MG cells. U87MG cells were treated with compounds (5 μM) or DMSO (control) for 24 h. After incubation time, tumor cells were employed for cell cycle analyses (panel A, B) and SA-β-Gal senescence marker (panel C). The cell cycle analyses was performed as describe in *Methods*. Representative cell cycle histograms of untreated and treated cells were shown (panel A). The data are presented as percentage of cell in the different phases (G0/G1, G2 or S) versus total cell number. Data represent the mean ± SEM of three different experiments. Statistical significance was determined with a one-way ANOVA with Bonferroni post-test: *P < 0.05 vs Control G0/G1 cells, #P < 0.05 vs Control G2 cells, §P < 0.05 vs Control S cells. (C) *Representative images of SA-β-Gal-expressing cells:* The panel shows the SA-β-Gal-expressing MDM2 inhibitor-treated and untreated cells at 24 h. (D) *Evaluation of apoptotic cells:* U87MG cells were treated with 5 μM Compound **1** or 10 μM Nutlin-3 for 4–16–24 h. After incubation time cells were collected and the phosphatydilserine externalization was evaluated using Annexin V protocol as describe in *Methods*. The data are expressed as percentage of apoptotic cells (Early-apoptotic in white, late-apoptotic/necrotic in grey) versus the total number of cells. Data represent the mean ± SEM of three different experiments. Statistical significance was determined with a one-way ANOVA with Bonferroni post-test: *P < 0.05, **P < 0.01, ***P < 0.001 vs Control. (E) *Relative mRNA quantification of PUMA:* U87MG cells were treated with Compound **1** or Nutlin-3 for 16–24 h. The relative mRNA quantification of PUMA was performed by real-time RT-PCR as describe in *Methods*. Data represent the mean ± SEM of three different experiments. Each experiment was performed in duplicate. Statistical significance was determined with a one-way ANOVA with Bonferroni post-test: **P < 0.01 vs Control.

p53 activation in proliferating cells often caused cell cycle arrest at the G1 or G2 phases, an event mainly involving the cyclin-dependent kinase inhibitor p21. According to these outcomes, cell cycle cytofluorimetric assay revealed a significant increase of DNA content in the G2 phase and a concomitant decrease in the G1 fraction in cells treated with compounds **1** and **7** (5 μM), suggesting a cell cycle block in G2 phase (Figure 7A and B).

At this step, a deeper biological characterization was performed on compound **1**. Following cell treatments with this compound, no activity of the senescence marker β-galactosidase was observed, indicating that an irreversible cell cycle block was not activated (Figure 7C). In addition, the treatment of U87MG cells with **1** (5 μM) was able to induce a significant phosphatidylserine externalization, both in the absence (early apoptosis), or in the presence of 7-amino-actinomysin binding to DNA (late apoptosis/death) (Figure 7D). Finally, a significant increase (approximately 4-fold) of PUMA mRNA level, a gene required for p53-controlled intrinsic apoptosis pathway, was evidenced (Figure 7E). All together these results indicated that compound **1** was able to activate apoptotic cell death in U87MG cells. In parallel, the standard MDM2 inhibitor, Nutlin-3, did not show any induction of specific apoptotic parameters at this short-time of treatment (24 h) (Figure 7D and E), in agreement with previous reports^15^,^16^.

### Δψm collapse induction and viability inhibition in U87MG cells

**Figure 8:**
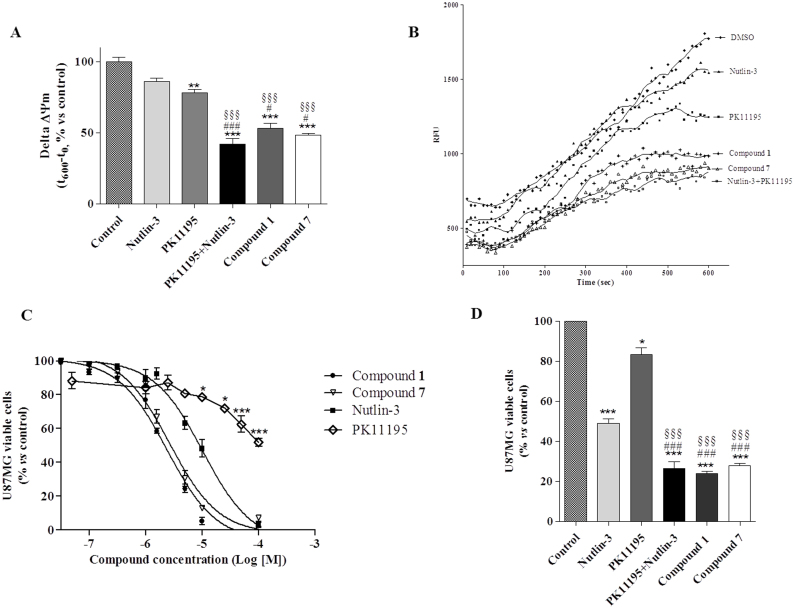
New synthesized compounds exert dissipation of *Δψm* and a dose dependent antitumoral effect intact U87MG cells. **(A) ***Evaluation of Δψm in U87MG cells:* U87MG cells were treated for 24 h with 5 μM compound **1**, or 5 μM compound **7**, or 10 μM Nutlin-3, or 10 μM PK1195, or a combination of Nutlin-3 and PK11195. After incubation time, mitochondria were isolated and the Δψm (for 5 μg of proteins) was evaluated using JC-1 protocol as describe in *Methods*. The data are expressed as the variation of JC1 uptake into mitochondria, calculated as the difference between RFU at the beginning and those read after 10 minutes. Data represent the mean ± SEM of three different experiments. Each experiment was performed in duplicate. Statistical significance was determined with a one-way ANOVA with Bonferroni post-test: **P < 0.01, ***P < 0.001 vs Control; §§§P < 0.001 vs PK11195 alone; #P < 0.05, ###P < 0.001 vs Nutlin-3 alone. (B) Representative graph of mitochondria potential evaluation using JC-1 protocol. The results were expressed as RFU units in time. (C), (D) *Evaluation of in vitro anti-proliferative effect:* the U87MG cells were treated with increasing concentrations of the compound **1**, **7**, PK11195 or Nutlin-3, or PK11195 (10 μM) and Nutlin-3 (10 μM) in combination, and the viable cells were counted after 24 h of treatment by Trypan blue exclusion assay. The data were expresses as percentage of compound-treated viable cells respect to control viable cells. Curves were generated using a sigmoidal dose-response curve model (GraphPad Prism 4 software) from which the IC_50_ values were derived. Data represent the mean ± SEM of three different experiments. Each experiment was performed in duplicate. Statistical significance was determined with a one-way ANOVA with Bonferroni post-test: *P < 0.05, ***P < 0.001 vs Control; §§§P < 0.001 vs PK11195 alone; ###P < 0.001 vs Nultlin-3 alone.

U87MG cells were treated with compounds **1** and **7** (5 μM) for 24 h, and then Δψm was measured. In mitochondria isolated from compound-treated cells, JC-1 accumulation was markedly reduced, demonstrating collapse of Δψm (Figure 8A and B). Of note, although it is difficult to make comparisons between different experimental settings, Δψm dissipation was higher when measured in mitochondria isolated from compound-treated cells with respect to that obtained in isolated treated mitochondria (compound **1**: Δψm dissipation vs control = 20.0 ± 1.8% and 47.0 ± 3.4% in isolated treated mitochondria and in intact cells, respectively; compound **7**: Δψm dissipation vs control = 43 ± 3.1% and 60.1 ± 3.2% in isolated treated mitochondria and in intact cells, respectively). These results suggest that amplification in Δψm collapse might be ascribable to the co-activation of TSPO and p53 pathways. To confirm these data, the effects of the standard MDM2 inhibitor, Nutlin-3, and of the TSPO ligand, PK11195, alone or in combination, were tested (Figure 8A and B). The results showed that 10 μM PK11195 alone was able to induce a significant Δψm dissipation, while 10 μM Nutlin-3 produced only a marginal effect after 24 h of cell treatment, in agreement with previous reports^15^,^16^. The percentage of Δψm dissipation elicited by compound **1** or **7**, tested at 5 μM, were significantly higher than those observed for PK11195 and Nutlin-3 alone. Moreover, the combination of the two standard drugs produced a synergic/additive effect on Δψm collapse (Figure 8A) comparable to that elicited by compound **1** or **7**, confirming that the simultaneous targeting of MDM2 and TSPO may cause an amplification response on MPTP opening.

Finally, to measure the ability of compounds **1** and **7** to affect cell survival, U87MG cells were treated with different compound concentrations, and then counted by conventional cell viability assays. The results showed that compounds **1** and **7** caused a dose-dependent inhibitory effect on U87MG cell survival (Figure 8C), with comparable IC_50_ values (2.5 ± 0.4 μM and 2.9 ± 0.5 μM for **1** and **7**, respectively). Also these experiments were repeated using Nutlin-3 and PK11195. Nutlin-3 caused a dose-dependent reduction of U87MG cell viability (Figure 8C), while PK1195 induced a significant inhibition of cell viability starting from 10 μM, and reached a maximal effect at 100 μM (inhibition at 100 μM: 52.0 ± 2.3% vs control), in line with literature data obtained in human cell lines^16^,^33^. As shown in Figure 8D, the two standard ligands alone, tested at 10 μM, caused a significant lower percentage of cell death with respect to compound **1** or **7**. However, when combined together, Nutlin-3 and PK11195 showed a synergic/additive effect on cell viability inhibition, comparable to that produced by treatment with **1** or **7** (Figure 8D), confirming that the simultaneous TSPO-p53 activation could be useful in blocking tumor cell proliferation.

## Discussion

The multi-target strategy has been assuming great importance in the therapy of GBM, a tumor for which the available treatments just offer a limited improvement in survival^4^,^5^,^6^. In terms of current multitherapy strategies, dual PI3K/mTOR inhibitors are emerging in GBM^34^. Several novel inhibitors dual targeting these kinases have been developed: XL765, for example, has demonstrated to reduce cell viability *in vitro*, and a possible effectiveness when combined with Temozolamide therapy^35^, confirming that drug combination or dual inhibitor might give improved results compared to monotherapy.

In this manuscript, for the first time, TSPO and p53 were considered as targets for combined therapy in GBM. Indeed, both TSPO activation and reactivation of p53 function, through the dissociation from its physiological inhibitor, MDM2, have largely been demonstrated to act as apoptosis inducers across multiple cell types, including glioma cells^8^,^11^,^12^,^15^,^16^. However, single molecules targeting both proteins at the same time are not known. In the present paper, we report that the simultaneous activation of TSPO and p53 by dual target single molecules, elicited antitumorigenic effects in GBM cells, improving outcomes over monotherapies by standard single-target agents.

In order to design a dual target single molecule, among a number of different chemotypes retained in our lab database as TSPO binders, we focused our attention on phenylindolylglyoxylamides (PIGAs). The indole is indeed considered a “privileged scaffold” for drug discovery, and PIGAs are prone to be easily substituted. Taking into account available SARs on TSPO, and the crystallographic data about the p53/MDM2 interaction, the 2-phenylindolglyoxylyldipeptide **1** and its acid analogue **7**, were rationally designed and synthesized (Figure 2). Docking studies confirmed the soundness of the design step (Figure 1).

The biological characterization of compounds **1** and **7** started with the evaluation of their ability to bind TSPO, and to induce, through TSPO activation, Δψm collapse in mitochondria isolated from GBM cells (Figure 3).

Then, the ability of **1** and **7** to bind MDM2, thus dissociating the MDM2/p53 complex, was investigated by NMR (Figure 4) and ELISA-based *in vitro* assays (Figure 5). Both techniques clearly demonstrated that both compounds **1** and **7** were able to efficaciously dissociate MDM2/p53 complex, with IC_50_ values in the nanomolar range. In particular, compound **1** appeared to be approximately 10 times more potent than the reference MDM2 inhibitors Nutlin-3 or ISA27^16^,^30^.

Subsequently, the cellular parameters commonly studied to support the reactivation of p53 pathway, following cell exposure with a MDM2 inhibitor^15^,^16^,^30^, were evaluated, and confirmed the expected mode of action of **1** and **7**. In fact, GBM cell incubation with new compounds led to a dose-dependent increase in p53 protein levels, due to a reduced interaction between p53 and MDM2, and a reactivation of p53 functions, as demonstrated by the enhancement of mRNA transcription levels of p53 target genes, MDM2 and P21 (Figure 6).

Interestingly, compound **1** induced both early and late apoptosis death of GBM cells, as well as the transcription induction of the pro-apoptotic gene PUMA, differently from what obserced with the standard MDM2 inhibitor, Nutlin-3, that did not show any induction of specific apoptotic parameters at short-time of treatment (Figure 7)^15^,^16^. These results support the hypothesis that compound **1** was able to trigger further intracellular pathways with respect to an agent specifically targeting MDM2/p53 interaction, such as Nutlin-3.

Finally, the ability of compounds **1** and **7** to modulate the cellular parameters which could be activated by both TSPO and p53, i.e. Δψm dissipation and cell viability, was examined in GBM cells and compared with the effects elicited by incubation with the single target standard ligands, Nutlin-3 and PK11195, used alone or in combination. Actually, the new compounds were able to induce an higher Δψm collapse with respect to that produced by Nutlin-3 and PK11195 incubated alone, and comparable to that raised by the combination of the two standards, confirming that the simultaneous targeting of MDM2 and TSPO cause an amplification response on MPTP opening (Figure 8A, B).

Concerning the viability assay, compounds **1** and **7** caused a dose-dependent inhibitory effect on U87MG cell survival, with comparable IC_50_ values, and percentage of cell death significantly higher with respect to Nutlin-3 and PK11195, singularly applied. When combined together, the two reference drugs showed a synergic/additive effect on cell viability inhibition, comparable to that elicited by our newly synthesized compounds **1** and **7**, confirming that the simultaneous TSPO/p53 activation could be useful in blocking tumor cell proliferation (Figure 8C,D).

Although additional experiments are required to study in deep this aspect, the overall effects on Δψm collapse and on GBM cell viability shown by compounds **1** and **7** could be reasonably ascribed to the simultaneous activation of TSPO and p53.

In conclusion, the use of this novel dual-targeted single-molecule could represent a promising approach to treat GBM, especially considering that the majority of GBM phenotypes maintains wild-type p53 and over-expresses TSPO and MDM2.

## Methods

### Molecular modeling

MDM2 structure selection. Several 3D structures of MDM2 can be found in the Protein Data Bank (PDB). Besides the apo-protein NMR structure (PDB code: 1Z1M) and the X-ray structure of MDM2 bound to the transactivation domain of p53 (PDB code: 1YCR), several complexes of MDM2 with different small molecules are reported. A superposition of all these structures on the alpha carbon atoms, using 1YCR as reference, shows that several of them, including 1YCR, lack the N-terminus residues 16–24 while others possess this region which forms (eg. 3LBL, 4HBM, 4DIJ, 4JVR, 4JVE, 1T4E, 4ERF, etc.) an ordered helix that not only modifies shape and size of the catalytic pocket but provides further points of interactions. Thus, among the non-truncated X-ray structures containing an organic compound, the one with the highest resolution (PDB code 3LBL, 1.60 Å) cocrystallized with a spirooxindole derivative was selected for docking studies. The binding modes of compounds **1** and **7** were studied by means of docking experiments with the new version of the widely-used AutoDock program (AutoDock4.2, AD4)^36^,^37^. The ligands 3D structures were created with the Maestro Build Panel (Maestro, Version 9.0.211. Schrodinger, L.L.C., New York, NY, 2009). The deprotonated state of **7** was confirmed by means of Marvin Sketch (ChemAxon). The target MDM2 structure was prepared through the Protein Preparation Wizard within the Maestro 9.0.2112 package using the OPLS-2001 force field. After removing water molecules and adding hydrogen atoms, the protein structures were converted to AD4 format files using ADT. The docking box has been centered on the H96 residue and AutoGrid4 was used to calculate grids points for all the ligand atom types (60 × 60 × 60 with 0.375 Å spacing). 100 separate docking calculations were executed for each ligand. Lamarckian genetic algorithm local search (GALS) method has been used for each docking calculation consisted of 25 × 106 energy evaluations. For each docking run the following parameters were used: population size of 150; 300 rounds of Solis and Wets local search were applied with a probability of 0.06; the mutation rate of 0.02; crossover rate of 0.8. The docking outcomes from each of the 100 calculations were clustered on the basis of the root-mean square deviation (RMSD 2.0 Å) between the Cartesian coordinates of the binder atoms and were ordered based on the free energy of binding. All figures were rendered using Chimera software package^38^.

### Chemical synthesis

#### General directions

A Reichert Köfler hot-stage apparatus was used to determine the melting points, that are uncorrected. For routine nuclear magnetic resonance spectra, compounds were dissolved in DMSO-*d*_6_ and a Varian Gemini 200 spectrometer operating at 200 MHz was utilized. Analytical TLC was carried out on Merck 0.2 mm precoated silica gel aluminum sheets (60 F-254). Optical rotatory powers ([α]D) were determined using a Perkin Elmer model 343 polarimeter, at a temperature of 22°C. The ≥95% purity of tested compounds was confirmed by combustion analysis. 2-Phenylindol-3-ylglyoxylyl chloride **6** was prepared according with reported procedures^17^,^18^.

#### N-Boc-L-leucine-L-phenylalanine ethyl ester 3

N-Boc-L-Leu **2** (0.208 g, 0.9 mmol) was solubilized in dry DMF (10 ml) in a nitrogen atmosphere; after addition of CDI (0.146 g, 0.9 mmol), the solution was stirred at room temperature for 2 h. Then, a solution of L-phenylalanine ethyl ester hydrochloride (0.413 g, 1.8 mmol) in 5 ml of the same solvent was added dropwise. The dipeptide **3** formed almost quantitatively within 4–5 h (TLC analysis). The solvent was evaporated under reduced pressure and the residue was triturated with saturated aqueous NaHCO_3_ solution, washed with water and filtered, yielding compound **3** in the desired purity degree. Yield 93%; mp = 78–80°C; [α]^22^_D_ = −19° (c = 2.0 mg/ml in MeOH).^1^H NMR (200 MHz, DMSO-*d*_6_) δ (ppm): 0.79–0.84 (m, 6H, 2CH_3_); 1.07 (t, 3H, *J* = 6.3 Hz, CH_3_); 1.34–1.55 (m, 12H, 3CH_3_, CH, CH_2_); 2.89–2.98 (m, 2H, CH_2_); 3.97–4.03 (m, 3H, CH, CH_2_); 4.40–4.46 (m, 1H, CH); 6.81 (d, 1H, *J* = 7.0 Hz, NH, exch. with D_2_O); 7.21 (s, 5H, Ar-H); 8.13 (d, 1H, *J* = 7.4 Hz, NH, exch. with D_2_O,). Anal. Calcd. for C_22_H_34_N_2_O_5_: C, 65.00; H, 8.43; N, 6.89. Found: C, 64.82; H, 8.22; N, 6.67.

#### L-leucine-L-phenylalanine ethyl ester 4

Trifluoroacetic acid (2 ml, 20 mmol) was added to a stirred solution of derivative **3** (0.122 g, 0.3 mmol) in 10 ml of CH_2_Cl_2_ and the mixture was maintained at room temperature for 6 h (TLC analysis). Evaporation of the solvent under reduced pressure yielded a solid residue that was taken up with water. The solution obtained was cooled in an ice bath, made alkaline (pH = 10) by adding solid K_2_CO_3_, and extracted with CH_2_Cl_2_. After drying with MgSO_4_, the organic solvent was eliminated under vacuum and product **4** was isolated in the desired purity degree. Yield 89%; mp = 268–270°C; [α]^22^_D_ = −7° (c = 2.0 mg/ml in MeOH).^1^H NMR (200 MHz, DMSO-*d*_6_) δ (ppm): 0.86–0.89 (m, 6H, 2CH_3_); 1.07 (t, 3H, *J* = 7.1 Hz, CH_3_,); 1.53–1.66 (m, 3H, CH, CH_2_); 3.01 (d, 2H, *J* = 7.4 Hz, CH_2_); 3.76 (t, 1H, *J* = 5.0 Hz, CH); 4.02 (dd, 2H, *J* = 7.2, 7.0 Hz, CH_2_); 4.50 (dd, 1H, *J* = 8.2, 8.6 Hz, CH); 7.26 (s, 5H, Ar-H); 8.25 (bs, 2H, NH_2_, exch. with D_2_O); 9.04 (d, 1H, *J* = 6.8 Hz, NH, exch. with D_2_O). Anal. Calcd. for C_17_H_26_N_2_O_3_: C, 66.64; H, 8.55; N, 9.14. Found: C, 66.44; H, 8.26; N, 8.90.

#### (2-phenylindol-3-yl)glyoxyl-L-leucine-L-phenylalanine ethylester 1

Oxalyl chloride (0.31 ml, 3.6 mmol) was added dropwise, at 0°C to a well-stirred mixture of the 2-phenylindole **5** (3.0 mmol) in freshly distilled diethyl ether (10 ml). The mixture was maintained at room temperature for 2–4 h. The generated precipitate was collected by vacuum filtration to give the acyl chloride **6** that was directly used in the subsequent reaction. A solution of compound **4** (0.685 g, 2.24 mmol) in 5 ml of dry toluene was added dropwise to a stirred suspension, cooled at 0°C, of the 2-phenylindol-3-ylglyoxylyl chloride **6** (2.0 mmol) in 15 ml of the same solvent, followed by the addition of a solution of triethylamine (0.34 ml, 2.4 mmol) in 1.5 ml of dry toluene. The reaction mixture was left under stirring for 12–24 h at room temperature (TLC analysis) and then filtered. The collected precipitate was washed with a 5% NaHCO_3_ aqueous solution and collected again to give a first portion of crude product. The toluene was removed under reduced pressure and the residue dissolved with CH_2_Cl_2_. The organic solution was washed with diluted HCl, a 5% NaHCO_3_ aqueous solution and water, dried (MgSO_4_), and evaporated to dryness to yield an additional amount of crude products. Compound **1** was finally purified by flash-chromatography (AcOEt/Ether petrol 60–80°C = 4/6 as eluent). Yield 74%; mp = 86–88°C; [α]^22^_D_ = −20° (c = 1.0 mg/ml in MeOH).^1^H NMR (200 MHz, DMSO-*d*_6_) δ (ppm): 0.75–0.82 (m, 6H, 2CH_3_); 1.01–1.32 (m, 6H, CH, CH_2_, CH_3_); 2.91–2.96 (m, 2H, CH_2_); 3.92–4.03 (m, 3H, CH_2_, CH); 4.41–4.47 (m, 1H, CH); 7.18–7.23 (m, 5H, Ar-H); 7.41–7.57 (m, 7H, Ar-H); 7.99 (d, 1H, *J* = 7.4 Hz, Ar-H); 8.39 (d, 1H, *J* = 7.4 Hz, NH, exch. with D_2_O); 8.57 (d, 1H, *J* = 8.6 Hz, NH, exch.with D_2_O); 12.34 (bs, 1H, NH, exch. with D_2_O). Anal. Calcd. for C_33_H_35_N_3_O_5_: C, 71.59; H, 6.37; N, 7.59. Found: C, 71.81; H, 6.11; N, 7.42.

#### (2-phenylindol-3-yl)glyoxyl-L-leucine-L-phenylalanine 7

Lithium hydroxide monohydrate (0.007 g, 0.3 mmol) was added to a suspension of (2-phenylindol-3-yl)glyoxyl-L-Leucine-L-Phenylalanine ethyl ester **1** (0.277 g, 0.5 mmol) in 20 ml of a MeOH/H_2_O (3:1) solution. The mixture was stirred under reflux overnight (TLC analysis). Subsequently, the solid precipitate was eliminated through vacuum filtration, and the solution was acidified with 10% HCl to pH = 5. Compound **7** was isolated directly in a pure state by filtration. Yield 70%; mp = 107–109°C, [α]^22^_D_ = −13.5° (c = 2.0 mg/ml in MeOH).^1^H NMR (200 MHz, DMSO-*d*_6_) δ (ppm): 0.71–0.83 (m, 6H, 2CH_3_); 1.23–1.45 (m, 3H, CH, CH_2_); 2.89–3.03 (m, 2H, CH_2_): 4.12–4.14 (m, 1H CH); 4.43–4.48 (m, 1H, CH); 7.19–7.59 (m, 13H, Ar-H); 8.01 (d, 1H, *J* = 7.4 Hz, Ar-H); 8.20 (d, 1H, *J* = 7.8 Hz, NH, exch. with D_2_O); 8.59 (d, 1H, *J* = 8.6 Hz, NH, exch. with D_2_O); 12.36 (bs, 1H, NH, exch. with D_2_O); 12.79 (bs, 1H, COOH, exch. with D_2_O). Anal. Calcd. for C_31_H_31_N_3_O_5_: C, 70.84; H, 5.94; N, 7.99. Found: C, 70.71; H, 6.05; N, 7.53.

### Competitive [^3^H]PK 11195 radioligand binding assay

Binding studies were performed as previously described^17^. Briefly, crude mitochondrial membranes, in 50 mM Tris-HCl pH 7.4 buffer, were incubated with 0.6 nM [^3^H]PK 11195 in the presence of new synthesized compounds (0.1 nM–10 μM) in a final volume of 0.5 ml for 90 min at 4°C. Incubations were terminated by rapid filtration through GF/C glass fiber filters and washed three times with 4 ml of cold buffer. The radioactivity was measured by liquid scintillation counter. Non-specific binding was estimated in the presence of unlabeled 1 μM PK 11195. IC_50_ value was determined using Graph-Pad Prism computer program (Graph Pad Software, version 5.0; San Diego, CA).

### Cell culture

The human glioblastoma multiforme U87MG cells were obtained from the National Institute for Cancer Research of Genova (Italy) and monitored for DNA profiling. The U87MG cells were cultured in RPMI medium supplemented with 10% FBS, 2 mM L-glutamine, 100 U/ml penicillin, 100 mg/ml streptomycin and 1% non-essential amino acids at 37°C in 5% CO_2_. The U87MG cells were plated at 5,000 cells/cm^2^. After 24 h, the culture medium was replaced with fresh medium containing newly synthesized compounds solubilized in DMSO for the indicated incubation times. DMSO was added to control cells (<1% v/v).

### Mitochondrial membrane potential (Δψm) dissipation analysis

The Δψm dissipation was assessed using the fluorescent dye 5,5′,6,6′-tetrachloro1,1′,3,3′-tetraethylbenzimidazolylcarbocyanine iodide (JC-1), that has been considered a more reliable and sensitive fluorescent probe for detecting differences in Δψm^39^. The JC-1 dye exhibits potential-dependent accumulation in mitochondria, where at higher concentration start to form J-aggregates indicated by a fluorescence emission shift from green (~529 nm, monomer emission maximum) to orange-red (~590 nm, aggregate emission maximum). Consequently, mitochondrial depolarization is indicated by a decrease in the orange-red fluorescence intensity. The JC-1 mitochondria staining procedure was performed preparing the JC-1 Working Solution (JWS) (0.2 mg/ml) in DMSO and keep the JWS on ice for 15–20 minutes to allow the dye to dissolve. Mitochondria were isolated from U87MG cells using the Mitochondria Isolation Kit (Sigma Aldrich, Milan, Italy) following manufacturer's instructions. For evaluation of newly synthesized compound effect on Δψm in isolated mitochondria, we added compounds **1** and **7** (5 μM) were added to the mitochondrial suspension for 15. Then, in a 96 well plate with black bottom, 90 μl of the JWS and 10 μl (equivalent to 5 μg of protein) of untreated or treated isolated mitochondria were added. The fluorescence (relative fluorescence units, RFU) of the sample was read in a spectrofluorimeter using a time-drive method (one acquisition each 10 sec, for 10 minutes), with settings excitation wavelength at 490 nm and emission wavelength at 590 nm. For evaluation of Δψm in whole cell, U87MG cells were treated with DMSO or compounds (new compounds at 5 μM and standard MDM2 and TSPO ligands at 10 μM), alone or in combination, for 24 h. After incubation time, cells were collected, mitochondria were isolated, and the JC-1 staining procedure was performed using the above-described method.

### NMR dissociation studies of recombinant MDM2/p53 complex

The recombinant human His-tagged N-terminal region of MDM2 (residues 1–118) was obtained using an Escherichia coli BL21(DE3) RIL expression system based on a pET-46Ek/LIC (Novagen) derivative vector in which the coding region of the MDM2 sequence was cloned. In particular, transformed cells were grown at 37°C in 1 L of LB medium up to 0.6 A_600_, induced for 3 h with 0.4 mM IPTG and collected by centrifugation. Cells were suspended with 5 ml of 20 mM TrisHCl pH 7.2, 20 mM β-mercaptoethanol, 1 mM PMSF and 1 mM EDTA (buffer A) and disrupted by a French press. The cell extract was then centrifuged at 13,000 rpm for 1.5 h to obtain the inclusion bodies as a pellet. This fraction was solubilised with 5 ml of 8 M urea in buffer A and centrifuged at 13,000 rpm for 1.5 h to remove insoluble material. MDM2 was purified under denaturing conditions by affinity chromatography, and to this aim, the supernatant was incubated over night at 4°C with 1 ml of NiNTA Agarose (Qiagen). After washing of the packed resin with 8 M urea in buffer A, pure MDM2 was eluted with 250 mM imidazole in the washing buffer. Fractions containing MDM2 were pooled together (4 ml) and refolded through three dialysis steps. The protein sample was firstly dialysed against 1 L of 2 M urea in 20 mM TrisHCl pH 7.2, 20 mM β-mercaptoethanol and 1 mM EDTA for 2 h at room temperature. A second dialysis step was carried out against 1 M urea in 20 mM TrisHCl pH 7.2 and 20 mM β-mercaptoethanol for 2 h at 4°C, and finally, the protein sample was dialysed over night at 4°C against 20 mM TrisHCl pH 7.2 containing 20 mM β-mercaptoethanol. The recombinant human His-tagged p53 protein (residues 1–312) was expressed and purified using the same protocol above mentioned for MDM2, except that the buffer used in the final dialysis contained also 0.2 mM ZnCl_2_. Purified proteins appeared homogeneous on 12% SDS-polyacrylamide gel electrophoresis, and the protein concentration was derived from absorbance readings at 280 nm, using a molar absorption coefficient (1 cm) of 0.54 and 0.69 calculated for MDM2 and p53, respectively, on the basis of their amino acid sequence.

NMR spectra were acquired at 25°C on a Varian Unity INOVA 700 MHz spectrometer equipped with a cryoprobe. NMR samples contained 0.1 mM of proteins in 20 mM TrisHCl, 150 mM KCl, pH 7.4, 5 mM β-mercaptoethanol, 0.02% NaN_3_. Water suppression was carried out by gradient echo^39^. NMR data were processed using the Bruker program BioSpin 3.0. For NMR ligand binding experiments, 300 μL of the protein sample containing 10% D_2_O, at a concentration of about 0.1 mM, and a 10 mM stock solution of compounds **7** and **1** in DMSO-*d*_6_ were used. The formal final molar ratio protein:inhibitor was 1:2. The addition of **1** from a stock DMSO solution (0.2 mM formal final concentration) to the MDM2/p53 complex caused sample precipitation indicating that the actual **1** concentration was lower than 0.2 mM.

### Dissociation studies of native MDM2/p53 complex

To test the ability of new compound to dissociate the native MDM2/p53 complex, a quantitative sandwich immune-enzymatic assay, on crude cell lysates obtained from U87 MG cells was developed. Cells were washed twice in ice-cold phosphate-buffered saline, collected by centrifugation, and suspended in lysis buffer (20 mM Tris HCl, 137 mM NaCl, 10% glycerol, 1% NONIDET^40^, 2 mM EDTA, pH 8) containing 1% of the Protease inhibitor Cocktail (Sigma Aldrich, Milan, Italy). Optimal composition of lysis buffer, as well as reaction conditions, were determined in preliminary experiments. 96-wells were pre-coated with a mouse full-length anti-MDM2 antibody (sc-965, Santa Cruz Biotechnology, 1:50 in 0.05% Poly-L-Ornithine) overnight at room temperature. Cell lysates (15 μg in a final volume of 100 μl) were pre-incubated with DMSO (control) or different compound concentration for 10 minutes at room temperature, and then transferred to the pre-coated wells for 60 min. After three quick washes with PBS/Tween 0.05% to remove unbound MDM2, each well was incubated for 15 min with 1% BSA, to block nonspecific sites, and then for 1.5 h at room temperature with a rabbit primary anti-p53 antibody (sc-6243, Santa Cruz Biotechnology, 1:250 in 5% milk). Then, wells were washed and incubated for 1 h with an anti-rabbit HRP-conjugate antibody (1:3000 in 5% milk), and washed again. The TMB substrate kit (Thermo Fisher Scientific) allowed a colorimetric quantification of the MDM2/p53 complex. Blanks were obtained processing cell lysates in the absence of the primary anti-p53 antibody. Absorbance's values at 450 nm were measured, background subtracted and sigmoid dose-response curves were generated using Graph Pad Prism 4 software, from which IC_50_ values of MDM2/p53 complex were derived. As standard controls, two characterized MDM2 inhibitors, Nutlin-3 and ISA27, were employed. The assay was also validated using different concentration of human recombinant p53 (Cat. #80-1892, Enzo Life Sciences)/MDM2 (Cat. #80-1894, Enzo Life Sciences) proteins. P53 and MDM2 standards were reconstituted and the MDM2/p53 complex was formed following the manufacturer's instructions. A semi-quantitative curve of the MDM2/p53 complex was obtained using different complex concentrations, ranging from 12.5/4 ng to 50/16 ng (p53/MDM2 ratio)^31^.

### U87MG cell viability assay

The effects of compound treatment on U87MG cell viability were evaluated by Trypan blue exclusion assays. Briefly harvested cells were mixed with an equal volume of 0.4% trypan blue dye. For quantization of cell viability, blue and bright cells were counted, and viability was calculated as the percentage of live (bright) cells versus control set to 100%. U87MG cells were treated with different concentrations (10 nM–10 μM) of each compound and after 24 h of incubation viable cells were counted. Sigmoid dose-response curves were generated from which the IC_50_ values were derived. PK11195 and Nutlin-3 were used in parallel as standard compounds.

### p53 stabilization analysis in U87MG cells

The western blot analysis for the evaluation of p53 protein levels was performed as previously described^16^. In brief, U87MG cells were treated with DMSO (control) or with different concentrations of the compounds for 12 hours, and then lysed for with RIPA buffer^16^. 30 μg of each sample was then resolved by SDS-PAGE (8.5%), transferred to PVDF membranes and probed overnight at 4°C with primary antibody anti-p53 (FL-393; Santa Cruz Biotechnology; 1:500). The primary antibody was detected using anti-rabbit IgG light chains conjugated to peroxidase (diluted 1:10.000). The peroxidase was detected using a chemioluminescent substrate (ECL, Perkin Elmer). Densitometric analysis of immunoreactive bands was performed using Image J Software.

The amount of MDM2/p53 complex was determined using co-immunoprecipitation experiments; U87MG cells were treated with DMSO (control) or 5 μM new compound for 8 h. One milligram of cell lysates, obtained as described above, was pre-cleared to precipitate and eliminate IgG. Samples were then incubated with an anti-MDM2 antibody (5 μg/sample) overnight, and then immunoprecipitated with protein A-Sepharose (2 h at 4°C). After washing, the immunocomplexes were resolved by SDS-PAGE (8.5%), transferred to PVDF membranes and probed overnight at 4°C with primary antibodies to p53 (FL-393, 1:500) or MDM2 (C-18, 1:500) as described above.

### Relative mRNA quantification of p53 target genes

The assessment of p53 target gene mRNA levels was performed by real-time reverse transcription polymerase chain reaction (real-time RT-PCR)^16^. In brief, total RNA was extracted from control cells as well as from cells treated with compounds (5 μM) for different hours, using Rneasy® Mini Kit (Qiagen, Hilden, Germany) according to manufacturer's instructions. Purity of the RNA samples was determined by measuring the absorbance at 260:280 nm. cDNA synthesis was performed with 500 ng of RNA using using i-Script cDNA synthesis kit (BioRad, Hercules, USA) following manufacturer's instructions. Primers used for RT-PCR were designed in intron/exon boundaries to ensure that products did not include genomic DNA (see Table S1). RT-PCR reactions consisted of 25 μL Fluocycle® II SYBR® (Euroclone, Milano, Italy), 1.5 μL of both 10 μM forward and reverse primers, 3 μL cDNA, and 19 μL of H_2_O. All reactions were performed for 40 cycles using 55°C as annealing temperature. The p53 target gene mRNA levels for each sample were normalized against β-actin mRNA levels, and relative expression was calculated by using Ct value. PCR specificity was verified by both the melting curve analysis and gel electrophoresis.

### Cell cycle and senescence analysis

The measurement of the percentage of cells in the different cell phases was performed using the Muse™ Cell Analyzer, Merck KGaA, Darmstadt, Germany). Briefly, U87MG cells were treated with DMSO or compounds (5 μM) for 24 h. Adherent cells were collected and centrifuged at 300 × g for 5 minutes. The pellet was washed with PBS and suspended in 100 μl of PBS; finally cells were slowly added to 1 ml of ice cold 70% ethanol and maintained o/n at –20°C. Then, a cell suspension aliquot (containing at least 2 × 10^5^ cells) was centrifuged at 300 × g for 5 minutes, washed once with PBS and suspended in the fluorescent reagent (Muse™ Cell Cycle reagent). After incubation for 30 minutes at room temperature in the dark, the measurements of the percentage of cells in the different phases was acquired.

The senescence marker SA-β-Gal was detected as previously described^16^ and cells were then washed in PBS (1×) and photographed at 100× magnification. Images of random light microscopic fields were captured (5 fields per well), and both blue and total cells were counted using ImageJ (ImageJ Software, version 1.41; USA).

### Annexin V and 7-AAD staining

Dual staining with Annexin V coniugated to fluorescein-isothiocyanate (FITC) and 7-amino-actinomysin (7-AAD) was performed using the commercially available kit (Muse Annexin V and Dead Cell Kit; Merck KGaA, Darmstadt, Germany). U87MG cells were treated with DMSO (control), Nutlin-3 (10 μM) or compounds (5 μM) for 4-16-24 hours. Both floating and adherent cells were collected, centrifuged at 300 × g for 5 minutes and suspended in cell culture medium. Then, a 100 μl aliquot of cell suspension (about 5 × 10^4^ cell/ml) was added to 100 μl of fluorescent reagent and incubated for 10 minutes at room temperature. After incubation, the percentages of living, apoptotic and dead cells were acquired and analyzed by Muse™ Cell Analyzer in accordance to the manufacture's guidelines. In cells undergoing apoptosis, annexin V binds to phosphatidylserine, which translocates to the outer leaflet of the cytoplasmatic membrane. Double staining is used to distinguish between viable, early apoptotic, and necrotic or late apoptotic cells^41^. Annexin V:FITC positive and/7-AAD positive cells were identified as early apoptotic. Cells which were Annexin V-FITC positive and 7-AAD positive were identified as cells in late apoptosis or necrotic.

### Statistical analyses

The nonlinear multipurpose curve-fitting program Graph-Pad Prism (GraphPad Software Inc., San Diego, CA) was used for data analysis and graphic presentations. All data are presented as the mean ± SEM. Statistical analysis was performed by one-way analysis of variance (ANOVA) with Bonferroni's corrected t-test for post-hoc pair-wise comparisons. P < 0.05 was considered statistically significant.

## Author Contributions

S.D., E.D.P., C.G., B.C. and M.L.T. performed most of the biological work. V.L.P., T.S. and L.M. conceived the idea and conducted the design and the synthesis. A.L. was in charge of expressing the MDM2 protein, while A.C. and A.L. performed the NMR experiments. E.B. and L.R. worked at the expression and purification of the MDM2 protein. L.M. and C.M. coordinated the project. L.M., V.L.P., C.M., F.D.S., A.C. and E.N. wrote the main manuscript text. All the authors reviewed the manuscript.

## Supplementary Material

Supplementary Information supplementary information
