# Supplementary material for: Apoptosis Therapy in Cancer: The First Single-molecule Co-activating p53 and the Translocator Protein in Glioblastoma
Source: Sci Rep. 2014 Apr 23;4:4749. doi: 10.1038/srep04749 (PMC3996484; doi:10.1038/srep04749)
Supplement: Supplementary Information [file srep04749-s1.pdf]

Apoptosis Therapy in Cancer: The First Single-molecule Co-activating p53 and the Translocator Protein in Glioblastoma

Simona Daniele[a], Sabrina Taliani[a], Eleonora Da Pozzo[a], Chiara Giacomelli[a], Barbara Costa[a], Maria Letizia Trincavelli[a], Leonardo Rossi[b], Valeria La Pietra[c], Luciana Marinelli[c*], Elisabetta Barresi[a], Alfonso Carotenuto[c], Antonio Limatola[c], Anna Lamberti[d], Ettore Novellino[c], Federico Da Settimo[a], Claudia Martini[a]

[a]Department of Pharmacy, University of Pisa, Pisa, Italy. [b]Department of Clinical and Experimental Medicine, University of Pisa, Italy. [c]Department of Pharmacy, University of Naples Federico II, Italy. [d]Dipartimento di Scienze Motorie, University of Naples "Parthenope", Naples, Italy.

Suppl. Figure 1

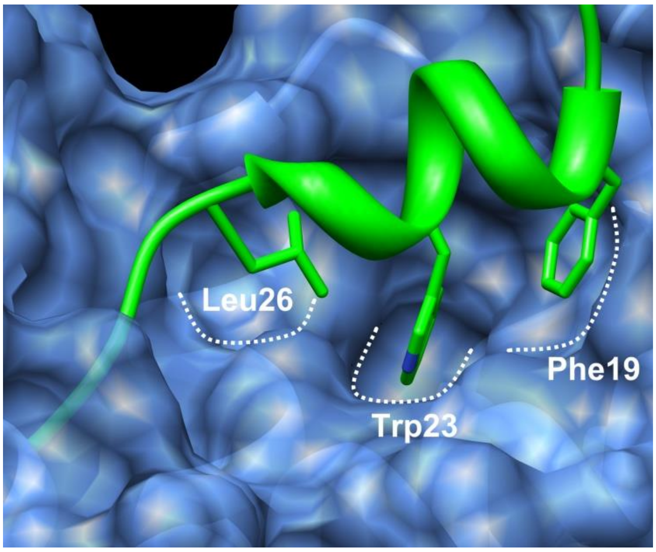

Supplementary Figure legends

Suppl. Figure 1. X-ray co-crystal structure of p53 (green) with MDM2 (cornflower), p53 residues and corresponding binding pockets in MDM2 are labelled in white.

Full-length blots relative to the cropped images showed in the main Figures

Figure 6

Figure 6 panel A

Left panel

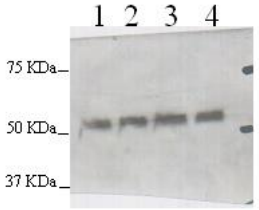

WB: anti-p53

Lanes: 1 = Control
2 = Compound **1** (1 μM)
3 = Compound **1** (5 μM)
4 = Compound **1** (10 μM)

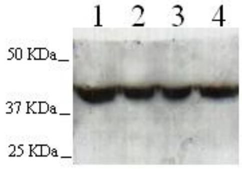

WB: anti-β-actina

Lanes: 1 = Control
2 = Compound **1** (1 μM)
3 = Compound **1** (5 μM)
4 = Compound **1** (10 μM)

Right panel

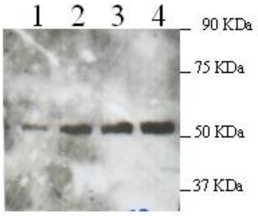

WB: anti-p53

Lanes: 1 = Control
2 = Compound **7** (1 μM)
3 = Compound **7** (5 μM)
4 = Compound **7** (10 μM)

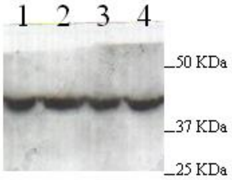

WB: anti-β-actina

Lanes: 1 = Control
2 = Compound **7** (1 μM)
3 = Compound **7** (5 μM)
4 = Compound **7** (10 μM)

Figure 6 panel C

Upper panel

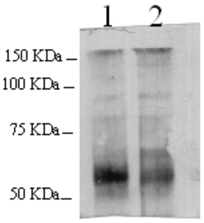

IP: anti-MDM2 WB: anti-p53
Lanes: 1 = Control
2 = Compound 1 (5 μM)

Lower panel

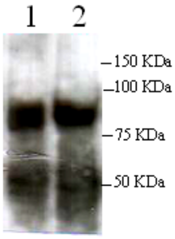

IP: anti-MDM2 WB: anti-MDM2
Lanes: 1 = Control
2 = Compound 1 (5 μM)

Supplementary Table 1. Real-time RT-PCR: primer nucleotide sequences, position in mRNA sequence and amplicon length.

Name and other designations	Accession number mRNA lenght	Primer nucleotide sequences	Primer position in mRNA sequence	Tm (°C)	Amplicon lenght
Mdm2, p53 E3 ubiquitin protein ligase homolog (mouse) (MDM2)	NM_002392.4 7472 pb	FOR: 5’-TCTAGGAGATTTGTTTGGCGT-3’ REV: 5’-TCACAGATGTACCTGAGTCC-3;	Exon 4 (548-568) Exon 6 (653-672)	60 60	125 pb
Cyclin-dependent kinase inhibitor 1A (p21, Cip1) (CDKN1A)	NM_000389 2175 pb	FOR: 5’-TGCCGAAGTCAGTTCCTTG -3’ REV: 5’-CATGGGTTCTGACGGACATC-3’	Exon 3 (50-68) Exon 4 (144-163)	60 62	134 pb
Bcl2 binding component 3 (BBC3), JFY1, PUMA	NM_001127240 1839 pb	FOR: 5’-GAGGAGGAACAGTGGGC-3’ REV: 5’- CTAATTGGGCTCCATCTCGG-3’	Exon 4 (652-668) Exon 5 (830-849)	56 62	198 pb
Tumor protein p53 (TP53), antigen NY-CO-13, cellular tumor antigen p53, p53 tumor suppressor	NM_001126112 2588 pb	FOR: 5’CTTTGAGGTGCGTGTTTGTG-3’ REV: 5’ GTGGTTTCTTCTTTGGCTGG-3’	Exon 8 (1006-1025) Exon 9 (1147-1166)	60 60	161 pb
Actin,beta (ACTB)	NM_001101.3 1852 pb	FOR: 5’-GCACTCTTCCAGCCTTCCTTCC-3’ REV-5’-GAGCCGCCGATCCACACG-3’	Exon 4 (862-884) Exon 6 (1097-1115)	70 62	254 pb
